# Comprehensive analysis of paraspeckle-associated gene modules unveils prognostic signatures and immunological relevance in multi-cancers

**DOI:** 10.1007/s12672-024-01188-6

**Published:** 2024-08-12

**Authors:** Zhuoyang Fan, Bowen Yin, Xiaochen Chen, Guowei Yang, Wei Zhang, Xiaodan Ye, Hong Han, Ming Li, Minfeng Shu, Rong Liu

**Affiliations:** 1 Department of Interventional Radiology, Zhongshan Hospital, Fudan University, Shanghai, 200032 China; 2 Shanghai Institute of Medical Imaging, Fudan University, Shanghai, 200032 China; 3 National Clinical Research Center for Interventional Medicine, Zhongshan Hospital, Fudan University, Shanghai, China; 4 Department of Pharmacology, School of Basic Medical Sciences, Shanghai Medical College, Fudan University, Shanghai, China; 5 Key Laboratory of Medical Molecular Virology, (MOE/NHC/CAMS), Shanghai Frontiers Science Center of Pathogenic Microorganisms and Infection, School of Basic Medical Sciences, Shanghai Medical College, Fudan University, Shanghai, China; 6 Clinical Research Institute, Shanghai Jiao Tong University School of Medicine, Shanghai, China; 7 Department of Radiology, Zhongshan Hospital, Fudan University, Shanghai, 200032 China; 8 Department of Cancer Center, Zhongshan Hospital, Fudan University, Shanghai, 200032 China; 9 Department of Ultrasound, Zhongshan Hospital, Fudan University, Shanghai, 200032 China; 10 Department of Thoracic Surgery, Zhongshan Hospital, Fudan University, Shanghai, 200032 China; 11 Department of Interventional Radiology, Xiamen Branch, Zhongshan Hospital, Fudan University, Xiamen, 361015 China

**Keywords:** Paraspeckle, Angiogenesis, Immune evasion, SFPQ, DDX39B, UBAP2

## Abstract

**Background:**

Hepatocellular carcinoma (HCC) is a leading cause of cancer-related deaths worldwide, characterized by high rates of angiogenesis and immune evasion. Paraspeckle genes, involved in gene regulation and RNA metabolism, have recently been linked to tumor progression. This study aims to elucidate the relationship between paraspeckle genes and HCC prognosis, focusing on SFPQ, DDX39B, and UBAP2.

**Methods:**

We analyzed HCC (LIHC) and prostate cancer (PRAD) samples from the TCGA database to explore the correlation between paraspeckle genes and angiogenesis. We conducted unsupervised clustering, risk scoring, and survival analysis to identify distinct patient groups and their clinical outcomes. Gene expression data were used to perform differential analysis and Gene Ontology (GO) enrichment.

**Results:**

Our analysis identified significant correlations between paraspeckle genes and angiogenesis across multiple cancer types. Elevated expression levels of SFPQ, DDX39B, and UBAP2 were associated with poor prognosis in HCC patients, and all of them has statistical significance. Unsupervised clustering of HCC samples based on paraspeckle gene expression revealed two distinct clusters, with high-risk patients exhibiting stronger immune suppression and tumor immune evasion. GO enrichment highlighted critical pathways related to angiogenesis and immune regulation. Additionally, a risk scoring model based on these genes effectively distinguished high-risk and low-risk patient groups, providing valuable prognostic insights.

**Conclusion:**

This study demonstrates that SFPQ, DDX39B, and UBAP2 are significantly associated with poor prognosis in HCC, likely due to their roles in promoting angiogenesis and immune suppression. These findings highlight the potential of paraspeckle genes as prognostic biomarkers and therapeutic targets, offering new avenues for personalized treatment strategies in HCC. Further research into their functional mechanisms and clinical applicability is crucial for advancing HCC treatment and improving patient outcomes.

**Supplementary Information:**

The online version contains supplementary material available at 10.1007/s12672-024-01188-6.

## Introduction

Hepatocellular carcinoma (HCC) is one of the most common types of primary liver cancer worldwide and poses a significant threat to human health. According to recent statistics, there are over 800,000 new cases of HCC annually, with particularly high incidence rates in Asia and Africa, highlighting the regional disparities in HCC prevalence [1]. The high incidence of HCC is associated with multiple factors. In East Asia, the primary factors include viral hepatitis (such as HBV and HCV infections), alcohol-related liver disease, and non-alcoholic fatty liver disease [2]. Due to the liver's substantial compensatory capacity, liver cancer often lacks noticeable symptoms in its early stages, resulting in most patients being diagnosed at advanced stages, which severely compromises prognosis and leads to high mortality rates [3]. Statistical data show that the five-year survival rate for HCC patients is less than 20%, with the average survival time for advanced-stage patients typically less than one year [4]. This underscores the significant challenges in HCC treatment and the urgent need for novel diagnostic and therapeutic approaches to improve patient survival and quality of life.

The biological complexity of HCC presents a major challenge in its treatment. As one of the largest internal organs, the liver has an extremely complex vascular structure, including a rich network of blood vessels and various cell types, providing ample blood supply to tumors growing within it [5]. The liver’s unique immune microenvironment, which includes diverse immune cells such as macrophages, natural killer cells, and T cells, further complicates HCC treatment due to the complex interactions within the tumor microenvironment [6]. Notably, tumor angiogenesis plays a crucial role in the growth and metastasis of HCC [7]. Angiogenesis not only supplies oxygen and nutrients to tumor cells but also promotes tumor progression by secreting various growth factors and signaling molecules [8]. Understanding the molecular mechanisms of angiogenesis has become a focal point, and many targeted therapies against angiogenesis in liver cancer have shown some efficacy, providing new avenues and strategies for HCC treatment [9].

Paraspeckles are a type of phase-separated structure within cells, functioning as membraneless organelles primarily involved in mRNA degradation and storage processes [10]. Previous research has demonstrated that phase separation in paraspeckles is vital for cellular function, particularly in regulating neuronal function and synaptic transmission [11]. Proteins and RNA within paraspeckles form dynamic aggregates through phase separation, participating in various crucial physiological processes within cells [12]. Recent studies also indicate that phase separation plays a significant role in tumor cells, affecting tumor cell growth and differentiation [13]. Our prior research has found that paraspeckles are involved in sublethal heat treatment of liver cancer, influencing tumor cell survival and proliferation by regulating cytokines and signaling pathways in the tumor microenvironment, providing new insights for liver cancer treatment [14].

HCC patients often exhibit immune evasion, further complicating treatment [15]. Immune evasion is the process by which tumor cells avoid immune surveillance and attack, playing a critical role in HCC progression [16]. During HCC development, tumor cells evade host immune surveillance through various mechanisms, including the expression of immunosuppressive molecules, secretion of immunosuppressive factors, and recruitment of immunosuppressive cells [17]. These mechanisms enable tumor cells to survive and proliferate within the host. The complex interactions within the tumor microenvironment, especially the potential role of paraspeckle genes in immune regulation, provide new perspectives for understanding tumor immune evasion. Research suggests that paraspeckle genes play a crucial role in regulating immune responses by modulating the expression of cytokines and immune receptors, thus affecting the function and activity of immune cells [18].

Against this backdrop, paraspeckle genes have garnered significant attention. Paraspeckle genes are involved in various biological processes, including gene regulation, RNA metabolism, and phase separation. Our research has revealed the relationship between paraspeckle genes and tumor development, highlighting their potential role in tumor angiogenesis and immune suppression. For example, paraspeckle genes such as SFPQ, DDX39B, and UBAP2 exhibit abnormal expression in various tumors, suggesting their important roles in tumorigenesis and progression. Therefore, this study aims to further investigate the relationship between paraspeckle genes and angiogenesis and immune regulation in HCC, providing new breakthroughs for HCC treatment. By elucidating the specific mechanisms of paraspeckle genes in liver cancer, we hope to identify new molecular targets for the diagnosis and treatment of HCC.

In this study, our analysis has identified paraspeckle genes such as SFPQ, DDX39B, and UBAP2 as potential biomarkers for HCC, aiding in the prediction of patient prognosis and offering new directions for personalized treatment strategies. By analyzing clinical data and gene expression profiles from a large cohort of HCC patients, we aim to clarify the expression patterns and biological functions of paraspeckle genes in HCC. Furthermore, through cell and animal model experiments, we will validate the specific mechanisms by which these genes influence HCC development and progression. By thoroughly investigating the functions and significance of paraspeckle genes in HCC, we aspire to contribute to the improvement of HCC treatment outcomes and provide more precise and effective therapeutic strategies for clinical practice.

## Materials and methods

### Dataset

RNA sequencing data for LIHC and PRAD patients were sourced from The Cancer Genome Atlas (TCGA) database via UCSC Xena (https://xenabrowser.net/datapage/), and the corresponding clinicopathological data were obtained from the cBioPortal website (http://www.cbioportal.org/).

*Inclusion Criteria*: Samples were selected based on the availability of complete RNA sequencing data and corresponding clinicopathological information. Specifically, we focused on hepatocellular carcinoma (LIHC) and prostate cancer (PRAD) samples to investigate the correlation between paraspeckle genes and angiogenesis.

### Somatic mutation and CNA analysis

The genetic alteration information for individuals with endometrial carcinoma was sourced in the “Mutation Annotation Format (maf)” from the TCGA GDC Data Portal. Genes associated with paraspekle were screened from R package ‘msigdbr’ and paraspeckle-related genes [19], and 41 genes were selected. To process and display the data related to the 41 mutated genes, we employed the “maftools” R package, along with the “ComplexHeatmap” tool. Tumor Mutation Burden (TMB) was calculated as the aggregate count of nonsynonymous mutations for each megabase of the coding area [20]. GISTIC 2.0 and GenePattern (accessible at https://www.genepattern.org/) were utilized to identify notable amplifications or deletions across the entire genome in relation to Copy Number Alterations (CNAs). The criterion set for identifying amplifications was a copy number exceeding one, while a copy number falling below -1 was used as the benchmark for detecting deletions.

### Unsupervised clustering for circadian rhythm genes

The paraspeckle-associated genes were sourced from the Molecular Signatures Database (MSigDB) and KEGG pathways. We meticulously curated and analyzed a set of 41 paraspeckle-related genes to identify distinct expression profiles. Using these profiles, we applied unsupervised clustering to categorize the various paraspeckle expression patterns within the TCGA data. To ensure robust and reliable classification, we utilized a consensus clustering approach implemented with the “ConsensusClusterPlus” package in R, performing 1000 iterations to guarantee the stability and reproducibility of the clusters.

Clustering Algorithms: Unsupervised clustering was performed using the “ConsensusClusterPlus” package in R, with the process repeated 1000 times to ensure the stability and reliability of the classifications.

### Weighted gene co-expression network analysis

In our research, we employed the Weighted Gene Co-expression Network Analysis (WGCNA) method using the “WGCNA” package in R to group gene modules that show the strongest correlation with the paraspeckle signature, as determined by single-sample Gene Set Enrichment Analysis (ssGSEA). We chose a soft-thresholding power (β) of 10 to create the adjacency matrix, which was achieved by elevating the Pearson correlation matrix of intergenic relationships to this power. Subsequently, to pinpoint candidate modules linked with the paraspeckle signature, we focused on modules that exhibited the most significant module-paraspeckle associations. In this context, Module Membership (MM) denoted the correlation between module eigengenes and individual gene expression patterns, whereas Gene Significance (GS) was quantified as the magnitude of the correlation between a gene and the clinical trait under study.

### Construction of riskscore with LASSO regression model

We conducted a Univariate Cox regression analysis to assess the association between overall survival (OS) and genes that were differentially expressed in the estrogen response pathway. Using a p-value threshold of less than 0.05, we applied the Least Absolute Shrinkage and Selection Operator (LASSO) method for Cox regression. This was executed using the “glmnet” package in R, with the penalty parameter determined through tenfold cross-validation. As a result of this process, 3 genes were identified as significant. The formula for calculating the risk score is as follows:

$$\text{Risk score} = \sum_{i=1}^{n} \text{Coef}_i \cdot x_i$$

(Coef_*i*_ means the coefficients, *x*_*i*_ is the FPKM value of each prognostic-related gene).

Based on the median value of the calculated risk scores, patients with Liver Hepatocellular Carcinoma (LIHC) in the Training, Validation, and Entire cohorts were stratified into two categories: low-risk and high-risk groups. To evaluate the effectiveness of this prognostic signature, we conducted time-dependent Receiver Operating Characteristic (ROC) analysis and Kaplan–Meier curve analysis. These analyses were instrumental in assessing the predictive accuracy of the risk score for survival outcomes and in comparing the survival probabilities between the low- and high-risk groups over time. The LASSO regression model was used for constructing the risk score system. The optimal cut-off point was determined using the 'survminer' package in R.

### Estimation of TME cell infiltration and signatures

The “ESTIMATE” algorithm was used to calculate Immune Score, and Stromal Score [21]. Furthermore, we used the ssGSEA (Single-sample Gene Set Enrichment Analysis) algorithm to quantify the relative abundance of each type of cells infiltrating the TME. The ssGSEA method was chosen in the process of signature score evaluation. TIP (Tracking Tumor Immunophenotype) is a meta-server that systematically integrates two existing third-party methods “ssGSEA” and “CIBERSORT” for tracking, analyzing, and visualizing the status of anti-cancer immunity and the proportion of tumor-infiltrating immune cells across a seven-step cancer-immunity cycle using RNA-seq or microarray data [22]. The correlations between the riskscore and the steps of the cancer-immunity cycle were analyzed using the “ggcor” R package.

### Statistical analysis

All statistical analyses in our study were conducted using R software, version 4.3.1, which can be accessed at http://www.R-project.org. For variables that followed a normal distribution, statistical significance was ascertained using Student's t-tests. Conversely, for variables not conforming to normal distribution, the Wilcoxon rank-sum test was the method of choice when making comparisons between two groups. Differences in survival were evaluated using Kaplan–Meier (K–M) curves, with the Log-rank test employed to determine statistical significance. In all instances, a p-value of less than 0.05 was the threshold for considering results as statistically significant.

## Results

**Fig. 1:**
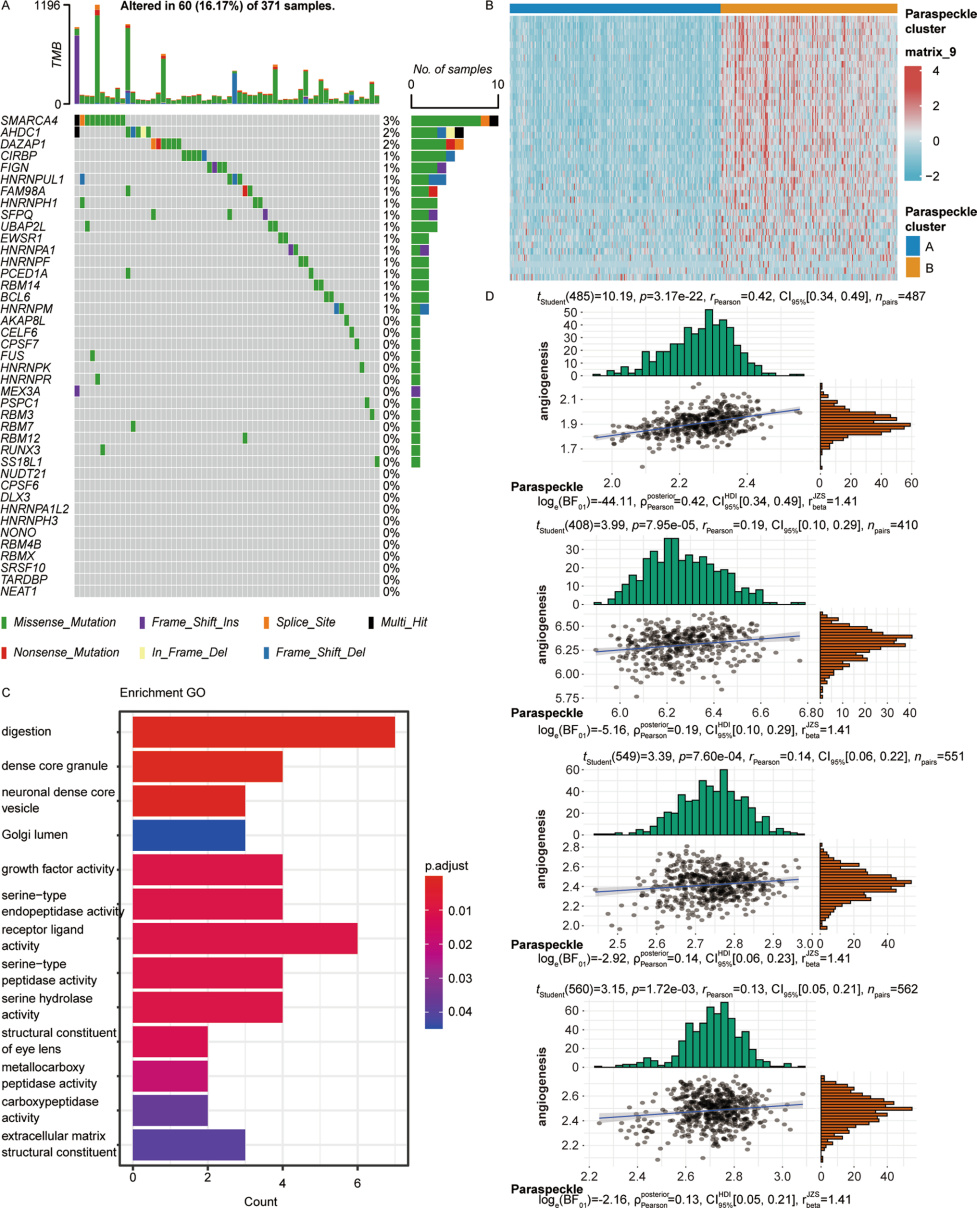
Correlation between paraspeckle and angiogenesis in various tumors in the TCGA database. **A** Mutation frequency of paraspeckle genes across 410 patients in the TCGA-LIHC (Liver Hepatocellular Carcinoma) cohort, depicted per individual patient. The upper histogram indicates the total number of mutations, while the right-side histogram shows the distribution of variant types. Numerical values above each segment of the right histogram represent the mutation frequencies for each variant type. **B** Unsupervised clustering of LIHC samples using the paraspeckle gene set, resulting in two distinct paraspeckle clusters A and B. **C** Differential gene expression analysis between paraspeckle clusters A and B, with Gene Ontology (GO) categorization. **D** Assessment of the correlation between the paraspeckle gene signature and angiogenesis signature in LIHC, LUAD (Lung Adenocarcinoma), PARD, and COAD (Colon Adenocarcinoma) tumors

Angiogenesis has consistently been one of the focal areas in cancer treatment [23]. In recent years, the paraspeckle has been identified as being closely linked to tumorigenesis and progression [24]. We aim to investigate the potential connection between these two phenomena. In our study on Liver Hepatocellular Carcinoma (LIHC), we conducted an extensive analysis of mutations and copy number variations (CNVs) in 41 paraspeckle genes across 371 samples. The results, depicted in Fig. 1A, revealed that among the most prevalent mutations in these genes, only SMARCA4 showed a significant mutant frequency of 3%, indicating that genetic mutations in paraspeckle genes may not be a major factor in angiogenesis perturbations in LIHC (Fig. 1A).

Additionally, through unsupervised clustering based on paraspeckle genes expression, we identified two distinct gene clusters in LIHC samples (Fig. 1B).

Differential gene analysis between these clusters, followed by Gene Ontology (GO) enrichment, identified critical pathways including digestion, neuronal dense core granule, and several activities related to serine peptidase and extracellular matrix structural constituents (Fig. 1C).

Moreover, our assessment of the correlation between the paraspeckle gene signature and angiogenesis across various cancer types (Fig. 1D)—including LIHC, Lung Adenocarcinoma (LUAD), PARD, and Colon Adenocarcinoma (COAD)—revealed a consistent positive correlation. In LIHC, the correlation was particularly strong (p = 3.17 × 10^−22^, r = 0.42), followed by LUAD (p = 7.95 × 10^−5^, r = 0.19), PARD (p = 7.60 × 10^−4^, r = 0.14), and COAD (p = 1.72 × 10^−3^, r = 0.13).

These findings suggest a significant association between paraspeckle gene expression and angiogenesis across these cancer types, providing new insights into the molecular mechanisms driving cancer progression.

**Fig. 2:**
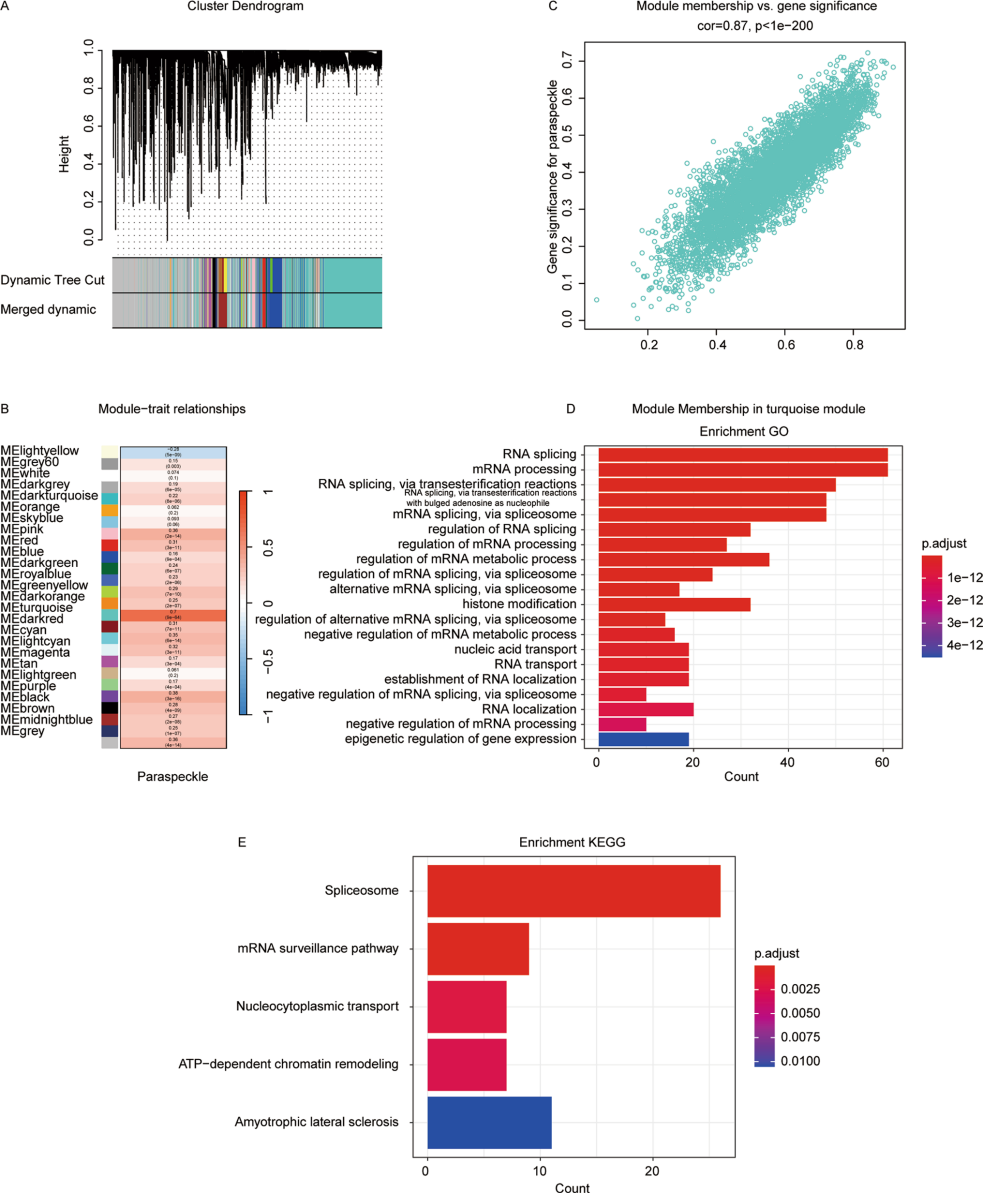
Identification and functional analysis of a gene co-expression module with shared paraspeckle characteristics in LIHC. **A** Selection of the top 25% most variable genes in LIHC for the construction of gene co-expression modules using Weighted Gene Co-expression Network Analysis (WGCNA), identifying 26 distinct modules. Application of WGCNA could be found in Method Part. **B** Application of the ssGSEA (single-sample Gene Set Enrichment Analysis) algorithm to calculate paraspeckle scores for each LIHC patient based on the paraspeckle signature. These scores were then correlated with each identified module. the Turquoise gene module exhibited the most significant correlation with the paraspeckle signature among the identified modules. **C** High correlation (r = 0.87) identified between the turquoise module genes and paraspeckle scores, indicating a significant association. **D** GO and **E** KEGG enrichment analysis of the turquoise module genes

We applied WGCNA to LIHC cohort generated from TCGA (Fig. 2A). β = 10 was selected to construct a standard scale-free network with the pick soft threshold function (Figure S1A), where genes were assigned to eight different modules using a cluster dendrogram.

To identify genes associated with paraspeckles, we correlated each module with paraspeckle traits, suggesting the turquoise module’s potential. The full module-trait correlation table is presented (Fig. 2B, S1B, S1C). Subsequently, the correlation analyses between turquoise module members and paraspeckle gene signature was conducted, revealing robust correlation (cor = 0.87, p < 1e−200) (Fig. 2C).

Furthermore, we performed the GO analysis, revealing that turquoise module genes were mainly enriched in functions such as RNA splicing, mRNA processing, RNA splicing via transesterification reactions, RNA splicing via transesterification reactions with bulged adenosine as nucleophile, and mRNA splicing via spliceosome (Fig. 2D).

Besides, KEGG analysis results raise the possibility that the turquoise module is a specific gene network regulating the pathway of Spliceosome, mRNA surveillance pathway, Nucleocytoplasmic transport, ATP-dependent chromatin remodeling, and Amyotrophic lateral sclerosis (Fig. 2E) and sharing similarities with the paraspeckle traits of LIHC.

**Fig. 3:**
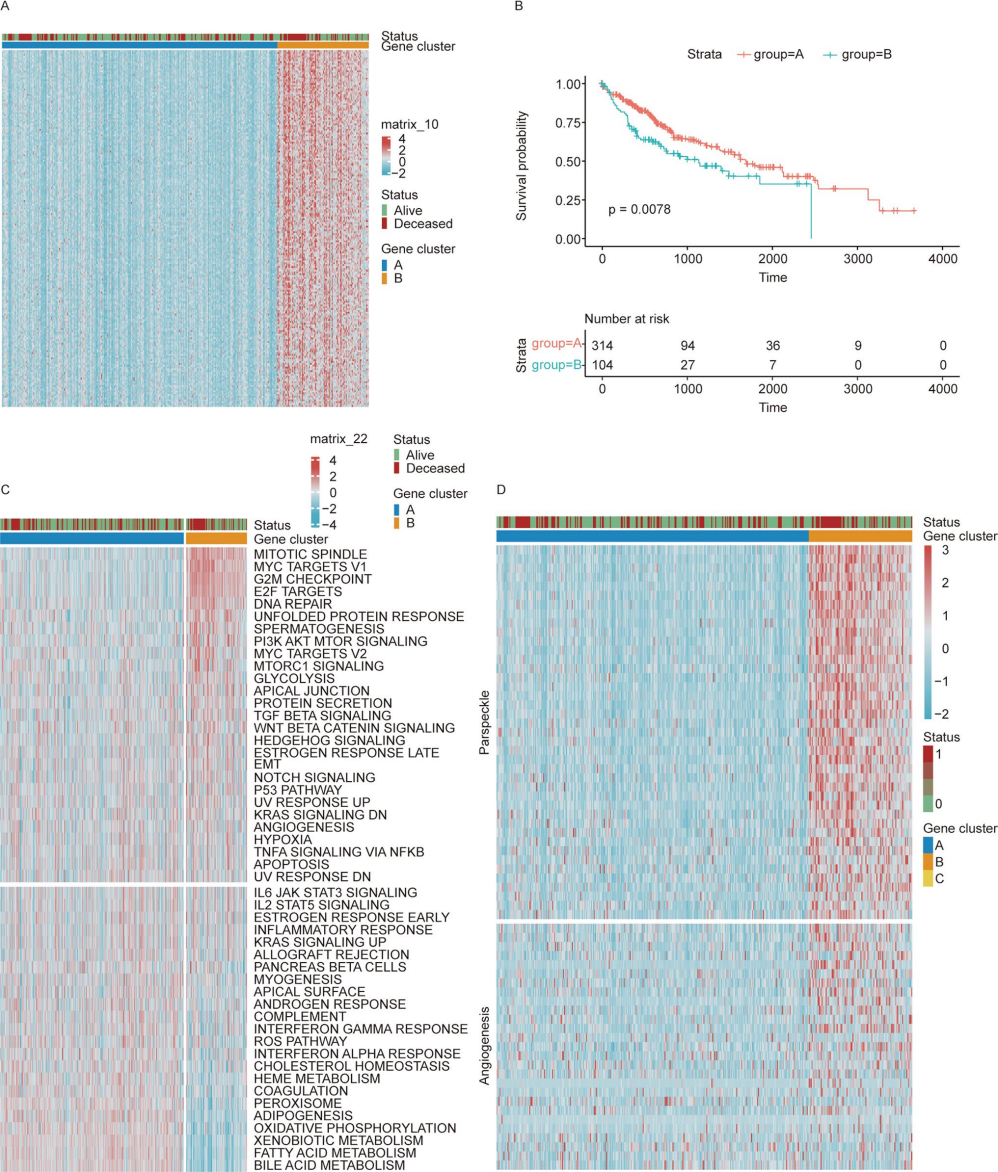
Discovery of two distinct gene clusters in the TCGA LIHC cohort mediated by turquoise module. **A** Unsupervised clustering analysis was performed on LIHC patients using genes from the turquoise module, resulting in two distinct clusters when k was set to 2. **B** Survival analyses for patient in two gene clusters using Kaplan–Meier curves. (Log-Rank test: p = 0.0078). **C** Application of the msigdbr package for ssGSEA (single-sample Gene Set Enrichment Analysis) on 50 Hallmark pathways (MSigDB), red represented the relatively high expression of corresponding pathway signature and blue represented the relatively low expression. **D** Heatmap analysis showed positive correlation between paraspeckle (para) and angiogenesis (angio) related genes

To further analyze the impact of genes in the turquoise module on patients with liver hepatocellular carcinoma (LIHC), we employed unsupervised clustering to divide LIHC patients into two distinct gene expression clusters based on the genes in the turquoise module (Fig. 3A).

Survival analysis of these clusters revealed significant differences in survival times between them (p < 0.05). Notably, patients in Cluster A exhibited significantly longer survival times compared to those in Cluster B (Fig. 3B).

Furthermore, we utilized the “msigdbr” package to analyze 50 cancer-associated hallmark pathways and conducted single-sample gene set enrichment analysis (ssGSEA) to compare the pathways between gene Clusters A and B. This analysis identified significant differences in pathway activation between the two clusters.

Specifically, pathways such as MITOTIC SPINDLE, MYC TARGETS V1, G2M CHECKPOINT, E2F TARGETS, DNA REPAIR, and UNFOLDED PROTEIN RESPONSE were significantly enriched in Cluster B. Conversely, Cluster A showed significant enrichment of pathways related to bile acid metabolism, fatty acid metabolism, xenobiotic metabolism, oxidative phosphorylation, adipogenesis, and peroxisome (Fig. 3C).

In Fig. 3D, our Heatmap analysis revealed that patients with lower expression of Paraspeckle also exhibit reduced expression of Angiogenesis; conversely, patients with higher expression of Paraspeckle demonstrate increased expression of Angiogenesis. A positive correlation between these two variables is evident.

**Fig. 4:**
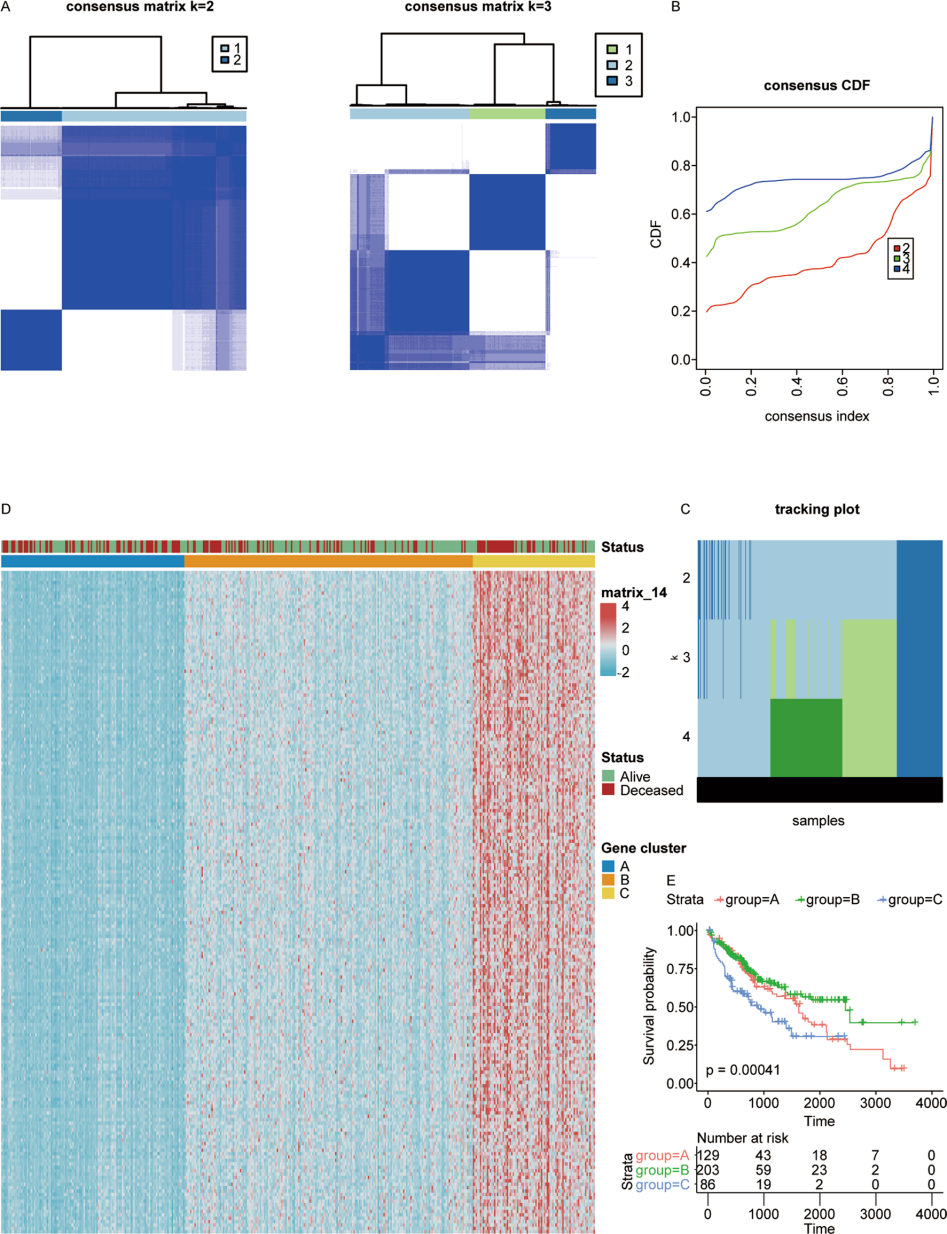
Elucidation of two gene clusters in the TCGA LIHC cohort through gene mediation. **A** The consensus matrices for the TCGA cohort were generated for values of k = 2 and 3. These matrices visually represent the degree of agreement among samples regarding their assignment to clusters for each specified value of k. Each element in the matrix reflects the proportion of resampling iterations in which a pair of samples clustered together. **B** A Cumulative Distribution Function (CDF) plot was constructed to depict the probability distribution of a real random variable. The plot was generated based on consensus scores for each k (ranging from 2 to 4), with each value represented by distinct colors, within TCGA)cohort. **C** Tracking plot depicting samples for different values of k (k = 2, 3, 4). **D** Unsupervised clustering analysis for LIHC patients using genes from the turquoise module, when k was set to 3. **E** Kaplan–Meier curves for OS of TCGA LIHC cohort when k = 3 (Log-Rank test: p = 0.00041)

To substantiate the reliability of our unsupervised clustering approach, we conducted a secondary verification by setting k = 3 (Fig. 4A–C), thus dividing the LIHC patients into three distinct groups (labeled as groups A, B, and C). This process is illustrated in Fig. 4D.

The outcomes of this classification revealed significant disparities in the survival periods among the three groups, as indicated by the p-values presented in Fig. 4E.

These results further validate the effectiveness of unsupervised clustering in stratifying LIHC patients based on their survival outcomes.

### Establishment of the LIHC prognostic signature risk score consisting of 3 genes

**Fig. 5:**
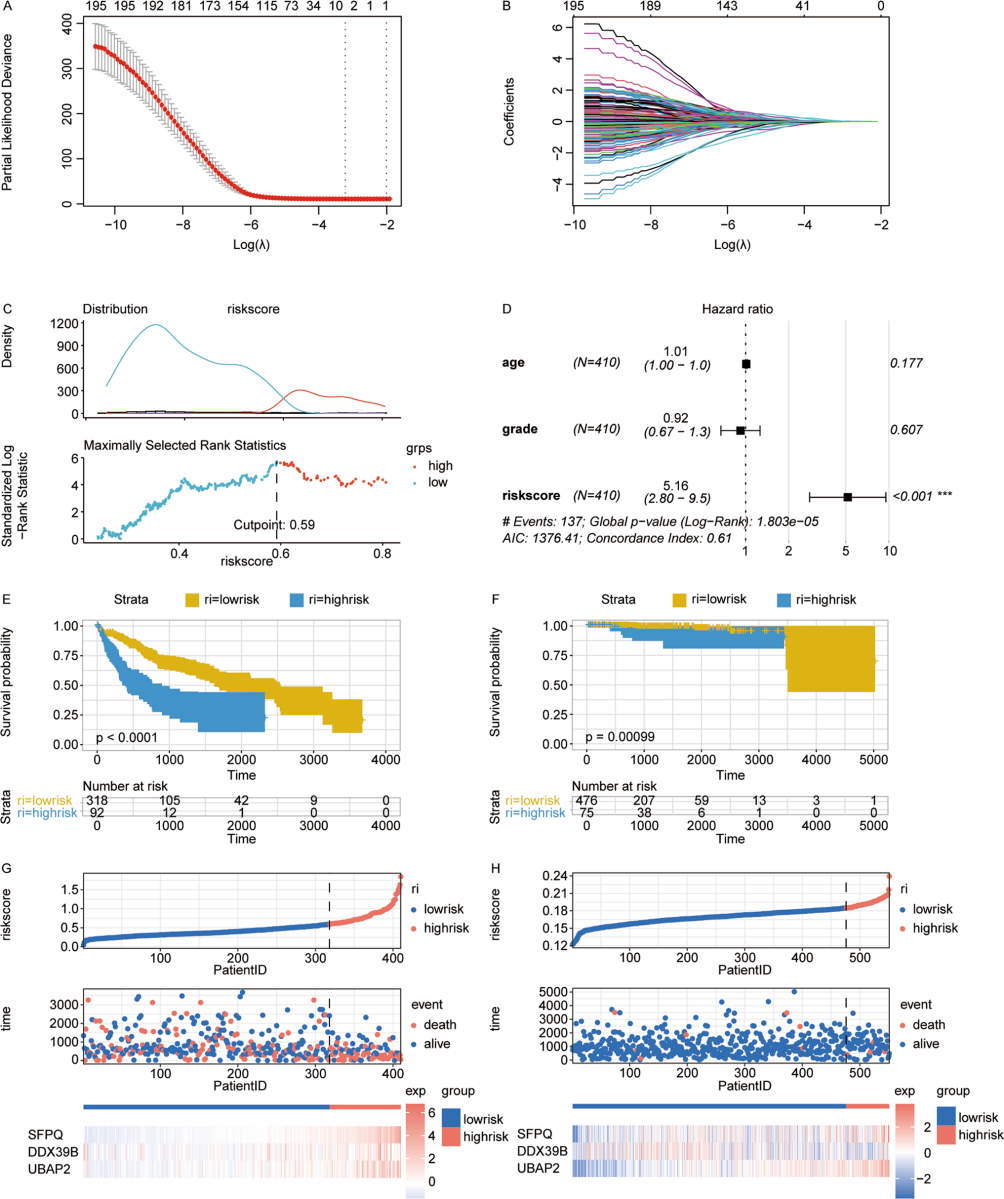
Development and evaluation of an immune-associated paraspeckle gene prognostic signature for overall survival in LIHC. **A** Robust prognostic genes identified through LASSO regression algorithm. **B** Distribution of LASSO coefficients in the tenfold cross validation. **C** Construction of a risk score system based on SFPQ, DDX39B, UBAP2, with a calculated cut-off point at 0.59, stratifying patients into high and low risk score groups. **D** Random Forest analysis depicting the importance of each variable in the model. The HR of riskscore is 5.16(2.80–9.5) p < 0.001. **E**–**G** Survival curves for LIHC and PRAD patients categorized into high and low risk score groups, illustrating significant survival differences along with the associated p-values (p < 0.0001;p = 0.00099). **H** Visualization of patients arranged according to their risk scores, correlating with their survival status and the expression patterns of the three identified genes

In our quest to identify key genes with potential prognostic value in Liver Hepatocellular Carcinoma (LIHC), we employed the least absolute shrinkage and selection operator (LASSO) regression algorithm. This method was instrumental in refining our search and pinpointing essential genes for further analysis (as illustrated in Fig. 5A and B). Through this process, we established a LIHC prognostic signature comprising three pivotal genes, followed by the calculation of the associated risk score. For validation purposes, the PRAD cohort was utilized.

Subsequently, we used the 'survminer' package to determine the optimal cut-point for categorizing LIHC patients into low- and high-risk groups (Fig. 5C and D).

Survival analysis further confirmed that a high risk score was significantly associated with reduced OS, a trend that was consistent across both the Training and Validation sets (Fig. 5E and F). We then conducted multivariate Cox regression analyses, incorporating various clinicopathologic parameters from the TCGA database. This comprehensive analysis highlighted a notable correlation between factors such as age, tumor grade, and the risk score in relation to overall survival.

We generated scatterplots and heatmaps to visualize the distribution of risk scores and their correlation with overall survival (OS), as well as the expression of prognostic risk genes across the entire patient group. These visualizations revealed that individuals with higher risk scores were more likely to have adverse outcomes (Fig. 5G and H).

**Fig. 6:**
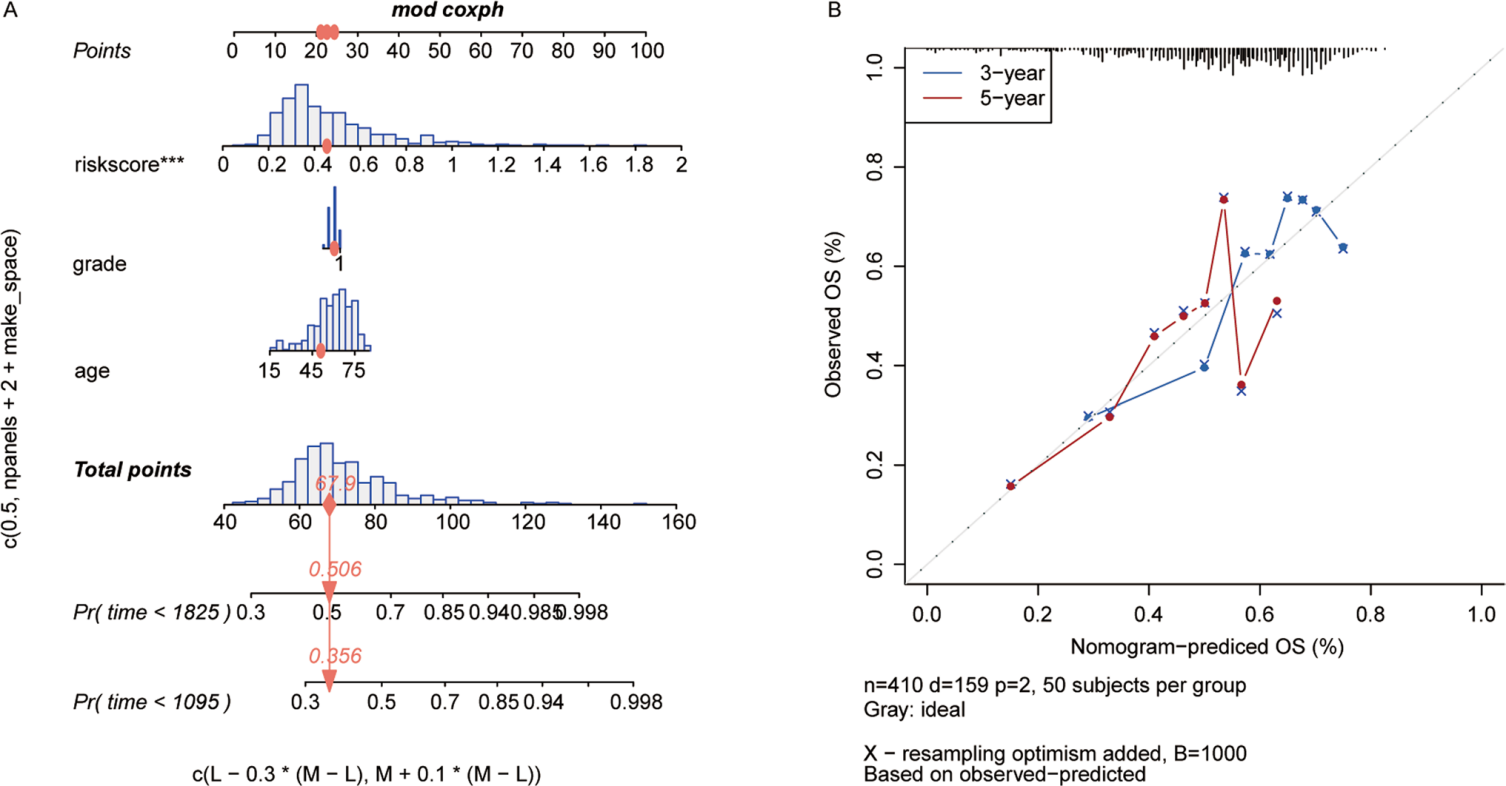
Construction of comprehensive predictive models for LIHC survival. **A** Development of a nomogram to forecast 3, 5-year overall survival rates in patients with LIHC. This graphical representation combines multiple prognostic factors to provide an individualized survival prediction. **B** Calibration curve assessing the accuracy of the nomogram in predicting 3, 5-year overall survival. The curve compares the predicted survival probabilities with the observed outcomes, with a high level of statistical significance (p < 0.001)

For patients with liver hepatocellular carcinoma (LIHC), we developed a predictive nomogram that utilizes risk score, tumor grade, and age from the entire dataset (TCGA-LIHC) to determine the likelihood of 3-year and 5-year overall survival (OS) as depicted in Fig. 6A. The effectiveness of the nomogram was demonstrated through calibration plots, with the ideal prediction shown by the 45° line in Fig. 6B. The outcomes indicate that the nomogram is effective in predicting survival.

### Exploration of immunological relevance in LIHC patients

**Fig. 7:**
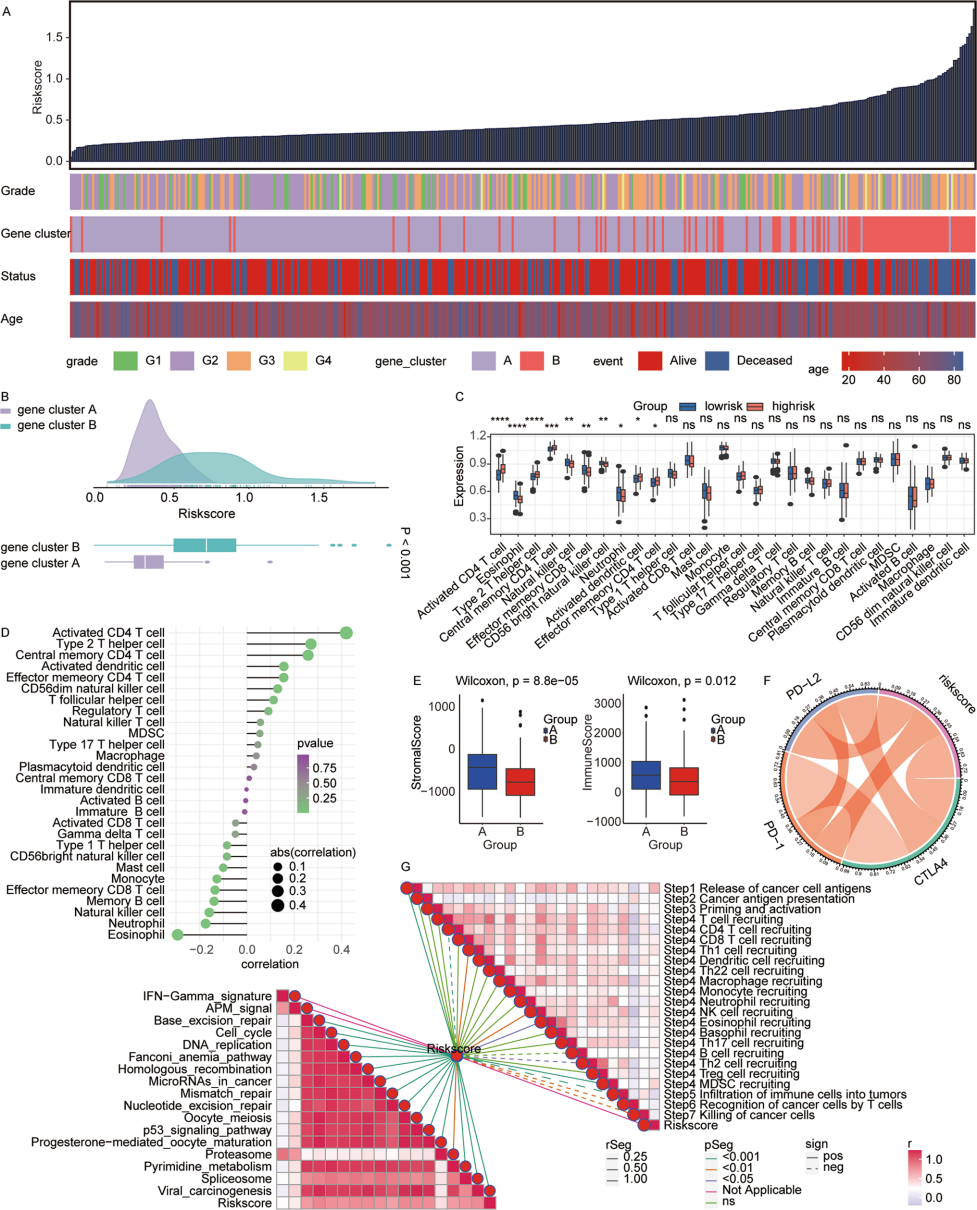
Correlation of risk score with the immune microenvironment in LIHC. **A** Analysis of the association between risk score and various clinical parameters including tumor grade,age and survival status in LIHC patients. **B** Comparison of risk scores between gene clusters A and B, highlighting a statistically significant difference (p < 0.001). **C** Examination of differences in the distribution of immune cell types between patients with high and low risk scores. **D** Correlation analysis of risk score and various immune cells in the LIHC microenvironment. The scores of immune cells were calculated by ssGSEA using corresponding immune cell signature. **E** Comparison of stromal and immune scores between gene clusters A and B. **F** Analysis of the relationship between risk score and immune checkpoint molecules including PD-L2, PD-1, and CTLA4. **G** Assessment of the risk score’s correlation with pathways related to tumor progression (lower left) and anti-tumor immune response steps (upper right) in LIHC

In an analysis stratified by risk scores among LIHC patients, we observed a clear relationship between higher risk scores and elevated tumor grades, which corresponded with lower survival rates (Fig. 7A). Furthermore, gene cluster B exhibited significantly higher risk scores compared to cluster A (Fig. 7B).

Using ssGSEA, we assessed the abundance of various immune cells and found statistically significant differences between high- and low-risk groups. The high-risk group displayed elevated levels of activated CD4 T cells, Type 2 T helper cells, central memory CD4 T cells, and effector memory CD4 T cells. Conversely, the low-risk group showed a higher abundance of natural killer cells, effector memory CD8 T cells, and CD56 bright natural killer cells (Fig. 7C). These results suggest that the high-risk group is more susceptible to a stronger immunosuppressive effect due to increased activated CD4 T cells and Type 2 helper T cells. In contrast, the elevated levels of natural killer cells and effector memory CD8 T cells in the low-risk group may offer protection against tumor growth.

Further correlation analysis indicated that risk scores were positively correlated with activated CD4 T cells, Type 2 T helper cells, and central memory CD4 T cells, while showing the strongest negative correlation with natural killer cells (Fig. 7D). Additionally, risk scores were significantly associated with immune checkpoint molecules PD-1, PD-L1, and CTLA4 (Fig. 7F), which potentially links paraspeckle gene expression to immune evasion in hepatocellular carcinoma. This association provides valuable insights into the development of personalized therapeutic strategies involving immune checkpoint inhibitors.

Furthermore, Group B had notably lower stromal and immune scores compared to Group A (Fig. 7E). Figure 7G (left) demonstrates a positive correlation between risk scores and tumor-associated pathways such as IFN gamma, APM signaling, and the proteasome. Meanwhile, Fig. 7G (right) reveals negative correlations between risk scores and immune cell infiltration into tumors, recognition of cancer cells by T cells, and the destruction of cancer cells, indicating a relationship between higher risk scores and immune suppression.

**Fig. 8:**
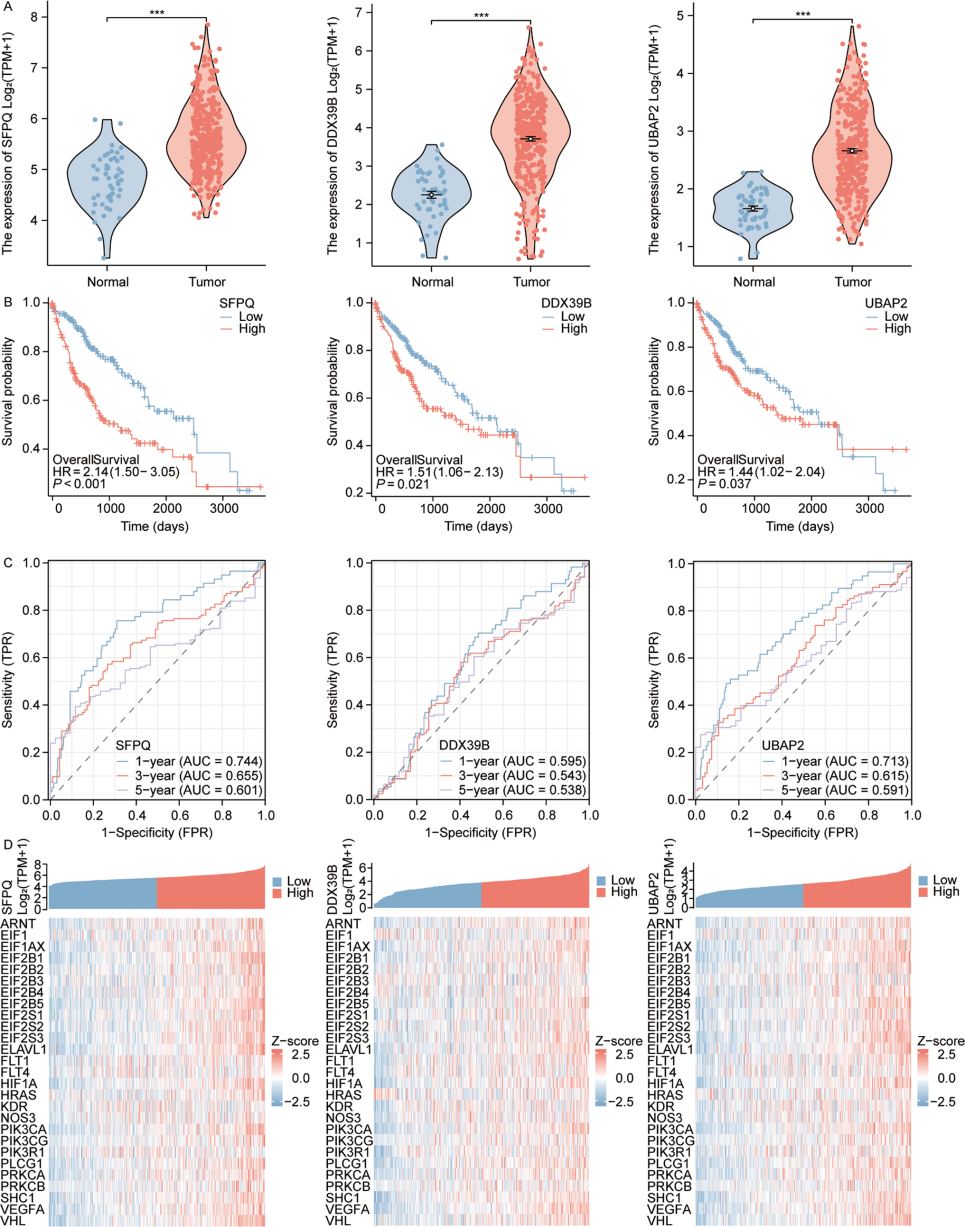
Association of candidate genes SFPQ, DDX39B, and UBAP2 with poor prognosis in liver cancer. **A** Elevated expression levels of SFPQ, DDX39B, and UBAP2 in liver cancer compared to adjacent non-tumor tissues, with statistically significant differences. **B** High expression of SFPQ, DDX39B, and UBAP2 in liver cancer correlates with poor prognosis. **C** Area under the Receiver Operating Characteristic (ROC) curves for SFPQ, DDX39B, and UBAP2 for 1, 3, and 5-year models in the validation dataset, indicating their predictive accuracy. **D** Positive correlation between SFPQ, DDX39B, and UBAP2 and genes associated with angiogenesis

Further analysis of the genes SFPQ, DDX39B, and UBAP2 demonstrated that their expression levels are significantly elevated in hepatocellular carcinoma tissues compared to adjacent non-tumorous tissues (p < 0.001), as depicted in Fig. 8A. High expression levels of these genes were found to be associated with poor prognosis, with hazard ratios (HR) of 2.14 (p < 0.001) for SFPQ, 1.51 (p = 0.021) for DDX39B, and 1.44 (p = 0.037) for UBAP2 (Fig. 8B). This highlights the significant association of SFPQ, DDX39B, and UBAP2 with poor prognosis in hepatocellular carcinoma patients, underscoring their potential value as prognostic markers. Additionally, Fig. 8D shows a positive correlation between these genes and angiogenesis-related genes, indicating their possible role in tumor angiogenesis and progression. ROC curve analysis further demonstrated that these genes are valuable in predicting patient outcomes, with the 1-, 3-, and 5-year AUC values exceeding 0.5. The 1-year AUC reached 0.744 for SFPQ and 0.713 for UBAP2, as shown in Fig. 8C. Moreover, these genes exhibited a positive correlation with paraspeckle-related genes (Fig. 8D), suggesting their involvement in this pathway.

## Discussion

In this study, we conducted a comprehensive analysis of hepatocellular carcinoma (HCC) and prostate cancer (PRAD) samples from the TCGA database, revealing a significant correlation between paraspeckle genes and tumor angiogenesis. High expression levels of SFPQ, DDX39B, and UBAP2 were found to be directly linked to poor prognosis in HCC patients. Through unsupervised clustering and risk scoring models, we effectively identified distinct patient groups with significant differences in gene expression, where the high-risk group exhibited stronger immune suppression and tumor immune evasion. The positive correlation of paraspeckle genes with tumor-related signaling pathways further underscores their roles in the tumor microenvironment and immune regulation. Additionally, the association of SFPQ, DDX39B, and UBAP2 with angiogenesis-related genes underscores their critical roles in tumor angiogenesis and progression, offering valuable insights for personalized treatment strategies and prognostic prediction in HCC and PRAD.

The results of this study show both consistencies and discrepancies with previous literature. Earlier research has indicated that paraspeckle genes play crucial roles in gene regulation and RNA metabolism [25], and are closely associated with tumorigenesis [26]. However, this study, through a comprehensive analysis of gene expression and immune characteristics in HCC, further elucidates the clear association between the paraspeckle gene cluster and tumor angiogenesis, immune suppression, and prognosis. Specifically, it highlights the correlation between the high expression of SFPQ, DDX39B, and UBAP2 and poor prognosis in HCC patients. Unlike previous studies, this research systematically validates the relationship between paraspeckle genes and the tumor immune microenvironment, clarifying how these genes accelerate tumor progression by promoting angiogenesis and immune suppression. This reinforces their potential value as diagnostic and therapeutic targets.

SFPQ, DDX39B, and UBAP2 demonstrate significant clinical relevance in this study. Their high expression is significantly associated with poor prognosis in HCC patients, and risk scoring analysis further indicates their efficacy in distinguishing between high-risk and low-risk patient groups. This endows them with potential value as prognostic biomarkers, aiding clinicians in more accurate stratification and prognosis assessment of HCC patients. Moreover, their positive correlation with genes related to angiogenesis suggests their roles in tumor angiogenesis, immune evasion, and progression. As key factors in angiogenesis and immune regulation, these genes hold promise as potential targets for future immunotherapies and anti-angiogenic treatments, offering new directions for personalized treatment of HCC. 

Despite revealing significant associations between paraspeckle genes, tumor angiogenesis, and immune regulation, this study has limitations. Firstly, the sample size is relatively limited, necessitating larger-scale studies to validate these findings' universality. Secondly, reliance on public database data may introduce biases or heterogeneity, affecting the generalizability of the results. In order to alleviate biases or heterogeneity,more cases from different datasets are need in future study. Future studies should also explore the specific mechanisms of paraspeckle genes across different tumor subtypes and in diverse patient populations to confirm our findings. Furthermore, the specific mechanisms of action of paraspeckle genes still require further exploration, particularly their functions across different tumor subtypes and interactions with other tumor-related signaling pathways. Future research directions should include multi-center clinical sample validation of these genes’ diagnostic and prognostic value, and employing single-cell sequencing and multi-omics technologies to delve deeper into the regulatory roles of paraspeckle genes in the tumor microenvironment, especially their specific molecular mechanisms in tumor immune evasion and angiogenesis. Exploring the potential roles of SFPQ, DDX39B, and UBAP2 in other cancer types will also provide more information for developing precise treatment strategies. On the other hand, different dimentions like in vitro, in vivo, ex vivo will be needed in future study to validate the potential roles of SFPQ, DDX39B, and UBAP2.

## Conclusion

This study systematically analyzed the complex relationship between paraspeckle genes, angiogenesis, and tumor immunity, determining that elevated expression of SFPQ, DDX39B, and UBAP2 is closely associated with poor prognosis in hepatocellular carcinoma (HCC) patients. Using a risk scoring model, we effectively distinguished between high-risk and low-risk patient groups, identifying more pronounced immune suppression phenomena in high-risk patients. The positive correlation between these genes and angiogenesis-related genes underscores their role in tumor angiogenesis and progression. This provides new insights into predicting HCC prognosis and developing personalized treatment strategies. Further research into their functional mechanisms and clinical applicability remains crucial for advancing HCC treatment and improving patient outcomes.

### Supplementary Information

Supplemantary file1 (DOCX 357 KB)

## Data Availability

Data is provided within the manuscript files, and is from TCGA datasets.
