# Supplementary material for: Comprehensive analysis of paraspeckle-associated gene modules unveils prognostic signatures and immunological relevance in multi-cancers
Source: Discov Oncol. 2024 Aug 12;15:345. doi: 10.1007/s12672-024-01188-6 (PMC11319543; doi:10.1007/s12672-024-01188-6)
Supplement: Supplementary file 1 — Supplemantary file1 (DOCX 357 KB) [file 12672_2024_1188_MOESM1_ESM.docx]

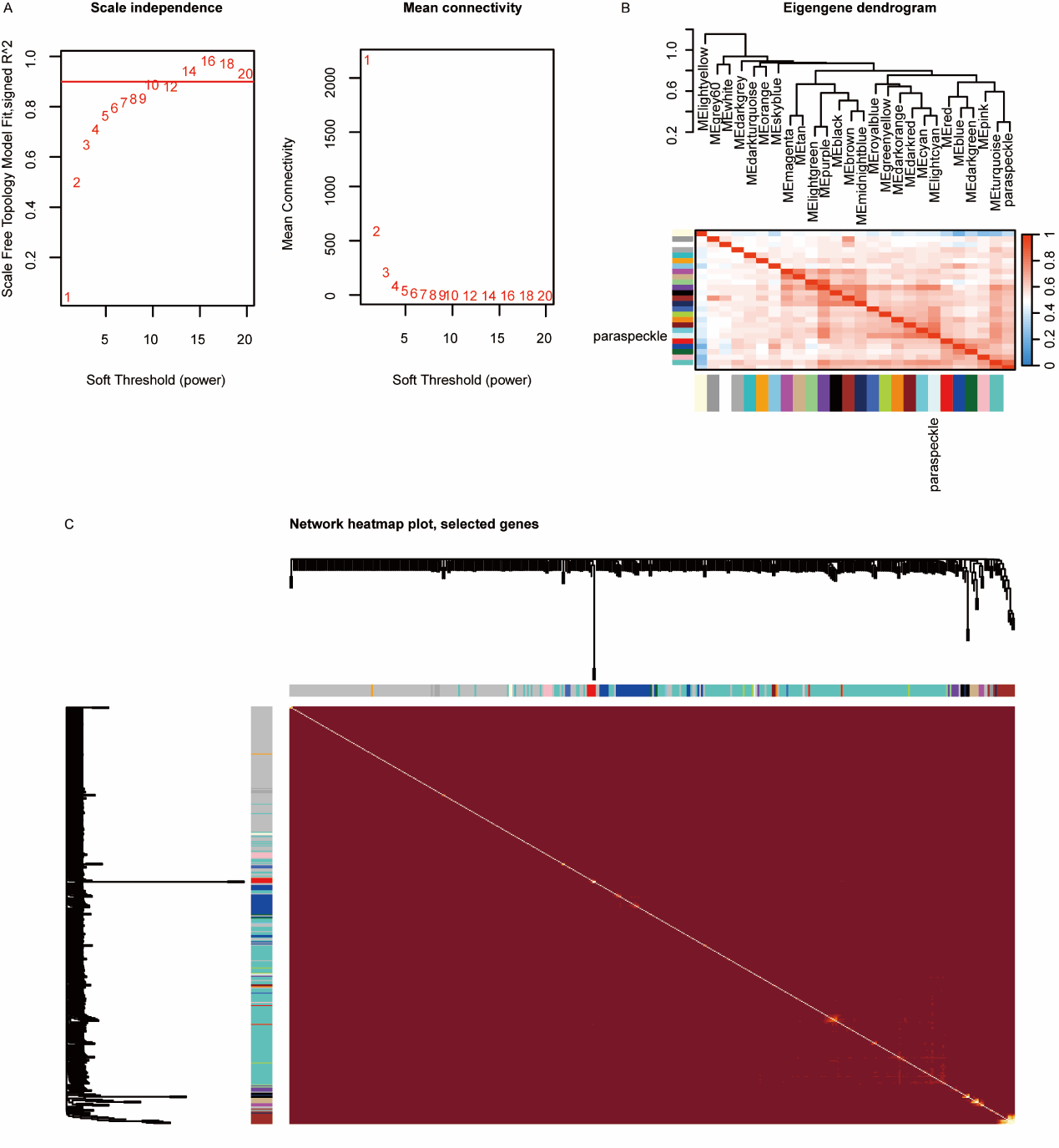


**Figure S1: Identification and functional interpretation of a gene co-expression module shared paraspeckle characteristics in LIHC.**

A. left, an assessment of the scale-free fit index was executed across a range of soft-threshold values (β).right, an analysis of the mean connectedness was performed for diverse soft-threshold values, aiming to discern optimal network properties.

B. Representation of the connectivity matrix, illustrating the strength of connections between different genes within the network. This matrix forms the basis for module detection.

C. Correlation analysis between paraspeckle-related genes and the identified gene co-expression modules. This graph highlights the strength and significance of associations between paraspeckle and each module.
